# Emotional Processing of Infants Displays in Eating Disorders

**DOI:** 10.1371/journal.pone.0113191

**Published:** 2014-12-02

**Authors:** Valentina Cardi, Freya Corfield, Jenni Leppanen, Charlotte Rhind, Stephanie Deriziotis, Alexandra Hadjimichalis, Rebecca Hibbs, Nadia Micali, Janet Treasure

**Affiliations:** 1 King's College London, Institute of Psychiatry, Department of Psychological Medicine, Section of Eating Disorders, London, United Kingdom; 2 University College London, Institute of Child Health, Behavioural and Brain Sciences Unit, London, United Kingdom; University of Udine, Italy

## Abstract

**Aim:**

The aim of this study is to examine emotional processing of infant displays in people with Eating Disorders (EDs).

**Background:**

Social and emotional factors are implicated as causal and maintaining factors in EDs. Difficulties in emotional regulation have been mainly studied in relation to adult interactions, with less interest given to interactions with infants.

**Method:**

A sample of 138 women were recruited, of which 49 suffered from Anorexia Nervosa (AN), 16 from Bulimia Nervosa (BN), and 73 were healthy controls (HCs). Attentional responses to happy and sad infant faces were tested with the visual probe detection task. Emotional identification of, and reactivity to, infant displays were measured using self-report measures. Facial expressions to video clips depicting sad, happy and frustrated infants were also recorded.

**Results:**

No significant differences between groups were observed in the attentional response to infant photographs. However, there was a trend for patients to disengage from happy faces. People with EDs also reported lower positive ratings of happy infant displays and greater subjective negative reactions to sad infants. Finally, patients showed a significantly lower production of facial expressions, especially in response to the happy infant video clip. Insecure attachment was negatively correlated with positive facial expressions displayed in response to the happy infant and positively correlated with the intensity of negative emotions experienced in response to the sad infant video clip.

**Conclusion:**

People with EDs do not have marked abnormalities in their attentional processing of infant emotional faces. However, they do have a reduction in facial affect particularly in response to happy infants. Also, they report greater negative reactions to sadness, and rate positive emotions less intensively than HCs. This pattern of emotional responsivity suggests abnormalities in social reward sensitivity and might indicate new treatment targets.

## Introduction

Eating Disorders (EDs) are complex disorders, characterised by problems with eating, weight, and body dissatisfaction. Difficulties with emotional processing have also been identified and have since been included as causal and maintaining factors of the illness in several new explanatory models [1]–[4]. Decreased emotional expression, and emotional avoidance/suppression are thought to be key features of Anorexia Nervosa (AN) [3]–[5]; and difficulties with trust and conflict have been highlighted in Bulimia Nervosa (BN) [5]. Broad anomalies in emotional processing, such as difficulties in emotion recognition, reduced facial communication, tendency to look away from emotional content, impaired abilities to read others' intentions as well as attachment insecurity have also been described in a recent meta-analysis in those with an ED [6].

Experimental studies from our group have investigated implicit and explicit components of social stimuli perception in EDs, such as attentional bias, emotional responsivity, and facial expression to positive and negative social displays. Findings indicated that participants with EDs have an attentional bias towards faces expressing rejection [7], social rank-related cues (i.e. dominance and submissiveness) [8], and a tendency to disengage from smiling, accepting faces [7]. Happy and sad faces did not produce a strong attentional response in this clinical population, but a trend for increased vigilance towards sad stimuli and disengagement from happy faces were found [9]. These data suggest that patients with EDs might be over-sensitised to socially evaluative threats.

In addition to anomalies in the perceptual aspects of emotional processing we have also found abnormal facial mimicry in response to videos of prototypical emotional displays, particularly in relation to joy and laughter [9]. Lack of facial affect and tendency to turn away were also seen in response to comic and tragic film clips [10].

The aforementioned studies investigated emotional processing in the context of adults emotional displays. In this study, we were interested in exploring the pattern of emotional processing in response to emotional displays of infants. Infant faces are considered to be particularly salient and rewarding for humans [11], [12]. Infant cues motivate adults to provide care through the activation of the reward system [13]. It has been suggested that the activation of motivational pathways that regulate approach responses to infant cues might be altered in mental disorders [16], [17]. For example, maternal depression has been associated with reduced amygdala activation to the positive emotion faces [14] and blunted responses to the distress faces of the mother's own infant [15], suggesting lower sensitivity to reward associated with these stimuli.

High levels of self-reported anhedonia have been found in people with EDs [18] and behavioural studies highlight attentional avoidance of compassionate adult faces [7] and reduced pleasure in response to amusing video clips [10]. The investigation of the response to infant cues in EDs might contribute to the understanding of the quality and extent of altered social hedonic processing in this condition, and inform the development of treatment modules to remediate these difficulties.

### Aims

The aim of this study was to experimentally investigate the different components of socio-emotional processing of infant cues in people with EDs. In particular, the following were measured: 1) attentional responses to photographs of happy and sad infants; 2) identification of and subjective emotional reactivity to happy, sad, and frustrated infant displays; 3) facial expressions in response to happy, sad, and frustrated (i.e. protest in response to physical constriction from an adult) infant displays.

### Hypotheses

We hypothesised that women with EDs compared to healthy controls (HCs) would:

1. Selectively attend to expressions of sadness and disengage from expressions of happiness during a visual probe detection task;
2. Accurately identify infants' emotions in short video clips, but rate negative emotions as more intense and positive emotions as less intense;
3. Report lower subjective emotional reactivity to video clips depicting positive and negative emotions;
4. Show fewer facial expressions in response to and higher tendency to turn away from video clips depicting positive and negative emotions.

## Methods

### Ethics statement

The investigation was carried out in accordance with the latest version of the Declaration of Helsinki. The study received ethical approval from King's College London (PNM/10/11-111) and all participants provided written informed consent after the nature of the procedures had been fully explained. No minors/children were included in the study.

### Participants

Participants were recruited from the Institute of Psychiatry Eating Disorders Unit's volunteer database, through advertisements placed on the BEAT (Beating Eating Disorders) website and through a circular email sent out to the staff and students at King's College London and University College London. Inclusion criteria consisted of: women aged between 18 and 55 years, fluent in English, with normal visual acuity and no motor impairment. A tailored version of the SCID-I (only the overview, screening and EDs modules, and open questions on past or present history of anxiety and mood disorders), which is a standardised interview for diagnostic assessment of DSM-IV disorders [19], was administered to screen for past or current mental health disorder in HCs and to confirm the diagnosis of EDs (i.e. Anorexia Nervosa, Bulimia Nervosa, Eating Disorders not Otherwise Specified).

### Measures

A demographic questionnaire including questions on: ethnicity, medication, visual impairment, neurological problems, employment status, current occupation, years of education, eating disorders duration, highest/current/lowest BMI, marital status, number of children, household sharing, diagnosis of psychiatric conditions in the family, and comorbidity was completed by all participants (Table 1). Participants also completed the following measures:

**Table 1:** Socio-demographic and clinical variables.

	AN	BN	HCs	Test statistic
Age	28.2 (10)	23.4(5.7)	26.4 (7.8)	F (2,112) = 1.5, p = NS
Years of education	16.3(2.8)	15.9 (2.3)	17.7 (2.9)	F (2,108) = 3.6, p = .03
				AN vs. HCs: p = .07
				AN vs. BN: p = NS
				BN vs. HCs: p = NS
Body Mass Index (Kg/m^2^)	15.9 (1.8)	21.8 (2.3)	21.9 (2.8)	F (2,105) = 72.3, p<.0001
				AN vs. HCs: p<.0001
				AN vs. BN: p<.0001
				BN vs. HCs: p = NS
Length of illness (months)	58.5 (80.7)	32.2 (44.0)	N/A	t(44) = 1.1, p = NS
Psychiatric medication (yes/no)	79.4%	75%	N/A	X^2^ = .1, p = NS
Previous hospital admissions (yes/no)	66.7%	54.5%	N/A	X^2^ = .5, p = NS
Psychiatric disorder other than ED diagnosed (yes/no)	38.2%	61.8%	N/A	X^2^ = 2.9, p = .09
Without a partner (single/divorced vs. in a relationship)	70.6%	58.3%	45.1%	X^2^(4) = 10, p = .04
				AN vs. BN = NS
				AN + BN vs. HCs: X^2^(2) = 7.8, p = .02
EDE-Q Restriction	3.7 (1.7)	3.8 (1.6)	.7 (.9)	F (2,113) = 79.9, p<.0001
				AN vs. HCs: p<.0001*
				AN vs. BN: p = NS
				BN vs. HCs: p<.0001*
EDE-Q Eating Concern	3.5 (1.2)	3.8 (1.3)	.2 (.4)	F (2,113) = 239, p<.0001
				AN vs. HCs: p<.0001*
				AN vs. BN: p = NS
				BN vs. HCs: p<.0001*
EDE-Q Weight Concern	3.9 (1.5)	4.7 (1.5)	.8 (.9)	F (2,113) = 107.6, p<.0001
				AN vs. HCs: p<.0001*
				AN vs. BN: p = NS
				BN vs. HCs: p<.0001*
EDE-Q Shape Concern	4.4 (1.6)	4.9 (1.3)	1.0 (1.0)	F (2,113) = 113.3, p<.0001
				AN vs. HCs: p<.0001*
				AN vs. BN: p = NS
				BN vs. HCs: p<.0001*
EDE-Q Total	3.9 (1.2)	4.3 (1.3)	.7 (.7)	F (2,113) = 161.8, p<.0001
				AN vs. HCs: p<.0001*
				AN vs. BN: p = NS
				BN vs. HCs: p<.0001*
DASS Stress	26.7 (10.1)	22.3 (11.8)	6.8 (5.9)	F (2,112) = 75.5, p = <.0001
				AN vs. HCs: p<.0001*
				AN vs. BN: p = NS
				BN vs. HCs: p = .002
DASS Depression	23.8 (13.1)	26.5 (12.1)	2.4(3.0)	F (2,112) = 98.2, p<.0001
				AN vs. HCs: p<.0001*
				AN vs. BN: p = NS
				BN vs. HCs: p<.0001*
DASS Anxiety	15.2 (10.9)	15 (9.2)	2.2 (3.2)	F (2,112) = 47.1, p<.0001
				AN vs. HCs: p<.0001*
				AN vs. BN: p = NS
				BN vs. HCs: p = .001*
	AN/BN (Combined)	HC		Test statistic
Closeness to children	65.2%	97.1%		X^2^(1) = 21.6, p<.0001
Closeness to adults	67.4%	84.2%		X^2^(1) = 4.5, p = .04
Unwanted sexual experiences before age 17	32.6%	12.9%		X^2^(1) = 6.6, p = .02
Physical abuse	18.6%	14.5%		X^2^(1) = .3, p = .6
Loss of parents	4.5%	5.8%		X^2^(1) = .08, p = 1.0
Early separation from parents (before age 17)	18.2%	9.1%		X^2^(1) = 2.0, p = .2
Attachment insecurity	39.8 (7.6)	27.2 (7.6)		t(59) = −6.3, p<.0001*
Attachment proximity	32.2 (5.9)	29.5 (5.8)		t(59) = −1.7, p = .09

Socio-demographic and clinical variables compared between groups, expressed as mean (standard deviation), and percentage. One-way ANOVAs followed by posthoc analyses, independent t-tests, and Chi-square tests calculated. Anorexia Nervosa  =  AN, Bulimia Nervosa  =  BN; Healthy Controls  =  HCs; NS  =  non significant.

*Significance after applying Bonferroni correction for multiple comparisons (Attachment insecurity and proximity: p< = .025; EDE-Q: p< = .01; DASS: p< = .016).

- Eating Disorder Examination Questionnaire (EDE-Q) [20]

This questionnaire is a 36 item self-report version of the original interview. The EDE-Q is composed of four subscales: weight concern, shape concern, eating concern, dietary restraint and a global score (a composite mean score of the four subscales). Scores ranging from 0 to 6 on a Likert scale correspond to the number of days over the past 4 weeks the respondent had experienced a specific attitude, feeling or behaviour. Previous studies show that the EDE-Q has high internal consistency [20] and moderate to high concurrent and criterion validity [21]. The Cronbach's alpha for the sample tested in this study was 0.92.

- Depression Anxiety Stress Scales (DASS) [22]

The DASS is a 21-item three-scale self-report measure of depression, anxiety, and stress. Higher scores are related to higher levels of depression, anxiety and stress. The scale has been validated and found to possess good reliability, with Cronbach's alpha to be 0.94 for Depression, 0.87 for Anxiety and 0.91 for Stress [23]. The overall Cronbach's alpha for the sample tested in this study was 0.97.

- The Childhood Experience of Care and Abuse Questionnaire (CECAQ) [24]

The CECA-Q assesses loss or separation from parents before the age of 17, close relationships with adults and children, physical punishment and unwanted sexual experiences. Two subscales measure parental antipathy and neglect (16 questions). The test-retest reliability has been found to be satisfactory for both subscales [24]. For the purposes of this study, data on parental antipathy and neglect were not included.

- Vulnerable Attachment Style Questionnaires (VASQ) [25]

The Vulnerable Attachment Style Questionnaire (VASQ) was developed to provide a brief self-report tool to assess adult attachment style. Items reflect behaviours, emotions and attitudes relating to attachment relationship style. The VASQ includes two factors: “insecurity” and “proximity seeking”. In the original study, the insecurity dimension had highest scores for those with Angry-dismissive and Fearful styles, whereas the proximity-seeking scale had highest scores for those with Enmeshed attachment style [25].

- Visual probe detection task

This test assesses attentional bias. It is a visual probe-detection task originally developed by Posner, Snyder and Davidson [26]. The participant's task is to respond to a probe stimulus that is initially hidden from view behind one of two stimuli. A fast reaction time (RT) suggests that attention has been directed to the stimulus that obscured the probe.

The stimuli used in this task were twenty-four photographs of infant faces obtained from a validated collection with approval from the author [11]. Twelve happy-neutral pairs and 12 sad-neutral pairs were repeated twice and presented in random order for each participant, for a total of 16 practice and 64 experimental trials.

Each trial started with a fixation point shown on the computer screen for 500 ms and then replaced by a picture pair which appeared for 500 ms. Immediately after the offset of each picture pair, a probe (either: or) was presented in the location of one of the pictures. The probe remained on the screen until the participant made a response by pressing the appropriate labelled key on the keyboard. Participants were instructed to indicate, as quickly and accurately as possible, which probe appeared on the screen after the presentation of the picture pair. The task was programmed using E-Prime psychology software (Psychology Software Tools, Inc., Pittsburgh, PA).

- Film Task

The film task was based on the methodology used by Davies and colleagues [10]. Participants watched four film clips of infants displaying: happiness, sadness, frustration, and neutral expressions. All the film clips were acquired from the internet and the clip depicting a frustrated infant was used with approval from the author [27]. The clips were rated by fifteen blind HCs. Each clip was approximately one minute in length. Following the method used by Davies and colleagues [10], the order of the film clips was fixed and based on the premise that negative affect has a more lasting carry over effect. Film clips were presented in the following order: ‘neutral’ (baseline), ‘happy’, ‘neutral’ (repeated), ‘frustrated’ and ‘sad’.

Participants rated the film clips according to: 1) valence and intensity of emotion displayed (i.e. “emotion identification”: 17 adjectives rated on a 5-point scale, Emotional Assessment Scale – EAS) [28]; and 2) valence and intensity of emotion experienced whilst watching the video-clip (“emotion experienced”: 22 adjectives rated on a 5-point scale, Positive and Negative Affect Scale- PANAS) [29]. Total scores were derived for “emotion identification” and “emotion experienced”. Higher scores represented more intense positive/negative emotions identified/experienced.

### Statistical analyses

Statistical analyses were carried out using SPSS version 20.0. One-way ANOVAs were used to compare scores between groups (HCs, AN, BN) on the demographic and clinical questionnaires, followed by posthoc comparisons for significant findings. Repeated measures ANOVAs were calculated to compare attentional bias to happy and sad facial expressions, ratings on the EAS and PANAS, and frequencies of positive and negative expressions, and of looking away between the EDs group and HCs, and followed by independent *t*-tests for significant findings. Bonferroni correction for multiple comparisons was applied (i.e. Attentional bias: p< = .025; EAS, PANAS, and frequencies of facial expressions and looking away: p< = .02). Comparisons between ED subgroups were not conducted due to lack of specific hypotheses on potential differences and the small sample size of the BN subgroup.

The attentional bias scores to happiness and sadness were calculated following the analytical plan of MacLeod and Mathews [30]. The RTs for the trials when the probe replaces the emotional picture (sad or happy; valid trials) were subtracted from the RTs for the trials when the probe replaces the neutral picture (invalid trials; attentional bias score  =  invalid trials – valid trials). Data from trials with errors were discarded.

The films were coded according to the Facial Expression Coding System (FACES) [31], as has been used in previous research from our lab [9]. Facial expression was defined as ‘a change from a neutral expression to a non-neutral expression and then back to a neutral expression’. An additional expression was considered if the initial facial expression did not return to a neutral expression or shifted to another affective facial display instead. Facial expressions were coded according to their valence, and frequency, intensity (4-point scale) and duration (seconds) were calculated. The frequency of looking away was also counted, but the duration was not measured. Thus, the total scores derived from coding were: (1) frequency of positive expressions, mean intensity, mean duration; (2) frequency of negative expressions, mean intensity, mean duration; (3) frequency of looking away. Two researchers who were blind to subject diagnosis (J.L. and C.R) rated the participants' facial expressions. Inter-rater agreement was high for coding of facial expression in response to infant faces (κ = .9) For the purpose of this study, only congruent facial displays were included in the analysis (i.e. frequency of positive expression to happiness; and frequency of negative expression to sadness and frustration). Following Davies and colleagues' procedure [10], the frequency of facial expression was used as the prime index of emotional expression as frequency, intensity and duration were all significantly correlated (correlations conducted separately for each video clip; *p*<.0001). Technical difficulties with the film task (e.g. participant moves out of head shot) or participant refusing to have videotape made resulted in loss of some data points (14.5%).

All effect sizes (*ESs*) were calculated using Cohen's *d* [32] and described as negligible ( = 0 and <0.15), small (≥0.15 and <0.40), medium (≥0.40 and <0.75), large (≥0.75 and <1.10), very large (≥1.10 and <1.45) and huge (>1.45) (Cohen, 1992). A *p* value of 0.05 was used for significance.

### Procedure

This study was carried out in a single 90-min. session. The SCID-I was administered at the beginning of the session, followed by the questionnaires, the visual probe detection task, and the film task.

During the film clips, participant's faces were recorded with their consent using a small video camera on a tripod behind the screen, following the methodology used by Davies and colleagues [10]. After watching each film clip participants rated how they thought the person in the film felt, using the EAS, and how they felt, using the PANAS. At the end of the session, height and weight measures were obtained in order to calculate the Body Mass Index (Kg/m^2^) by the experimenter.

## Results

### Socio-demographic and clinical variables

One hundred and thirty-eight female participants were included in the study: 73 HCs and 65 individuals with an ED. Of those with an ED, 49 had Anorexia Nervosa (AN, *N* = 31 inpatients, *N* = 18 outpatients), and 16 individuals had Bulimia Nervosa (BN; *N* = 3 inpatients, *N* = 13 outpatients). The demographic and clinical questionnaires were completed by 79% of the sample (table 1). Participants in the ED and HC groups did not differ significantly in age, but HCs had more years of education. Participants with AN had a significantly lower BMI than people with BN and HCs. Overall, people with an ED (AN or BN) showed significantly higher levels of ED symptoms and depression, anxiety, and stress than HCs on the EDE-Q and DASS questionnaires. People with AN and BN did not differ significantly in length of illness, and frequency of comorbidity and admissions. In relation to social functioning, fewer people in the ED group reported to be in a relationship than HCs. People with an ED also reported significantly higher levels of attachment insecurity, less closeness to other children and adults during their childhood, and more often unwanted sexual experiences than HCs. There were no significant differences between samples in self-reported attachment proximity, and experiences of physical abuse and loss and separation from parents.

### Emotion perception: attentional bias to photographs of happy and sad infants

There were no significant differences between EDs and HCs in the attentional responses to pictures of happy and sad infants [Group: *F* (1, 136) = .4, *p* = .5; Group x Emotion: *F* (1, 136) = .9, *p* = .3]. However, mean scores (table 2) indicated that subjects with EDs disengaged from happy displays, whereas HCs showed an opposite pattern (i.e. attentional bias; *ES*  = 0.2). Both groups had an attentional bias towards pictures of sad infants.

**Table 2:** Means and standard deviations for: attentional bias, Emotional Assessment Scale (EAS), Positive and Negative Affect Scale (PANAS), facial expressions, and frequencies of looking away in the Eating Disorders (EDs) and Healthy Controls (HCs) samples.

	Eating Disorders sample	Healthy Controls sample
Attentional bias: happiness	−7.1 (59.8)	2.6 (50.9)
Attentional bias: sadness	5.6 (44.1)	4.0 (47.9)
EAS: happiness	13.1 (4.1)	14.7 (3.7)
EAS: frustration	17.5 (8.0)	18.1 (7.8)
EAS: sadness	14.0 (7.7)	11.9 (6.7)
PANAS: happiness	12.6 (8.2)	13.6 (7.0)
PANAS: frustration	9.0 (7.7)	7.5 (7.0)
PANAS: sadness	7.6 (6.3)	3.7 (4.6)
Facial expressions: happiness	1.4 (1.1)	2.8 (1.8)
Facial expressions: frustration	.8 (.9)	.9 (.7)
Facial expressions: sadness	.6 (.7)	.8 (.8)
Looking away: happiness	1.3 (2.8)	.7 (1.8)
Looking away: frustration	1.9 (3.3)	1.0 (2.3)
Looking away: sadness	1.7 (3.5)	1.1 (2.4)

### Emotion identification of infant happy, frustrated, and sad displays

**Figure 1:**
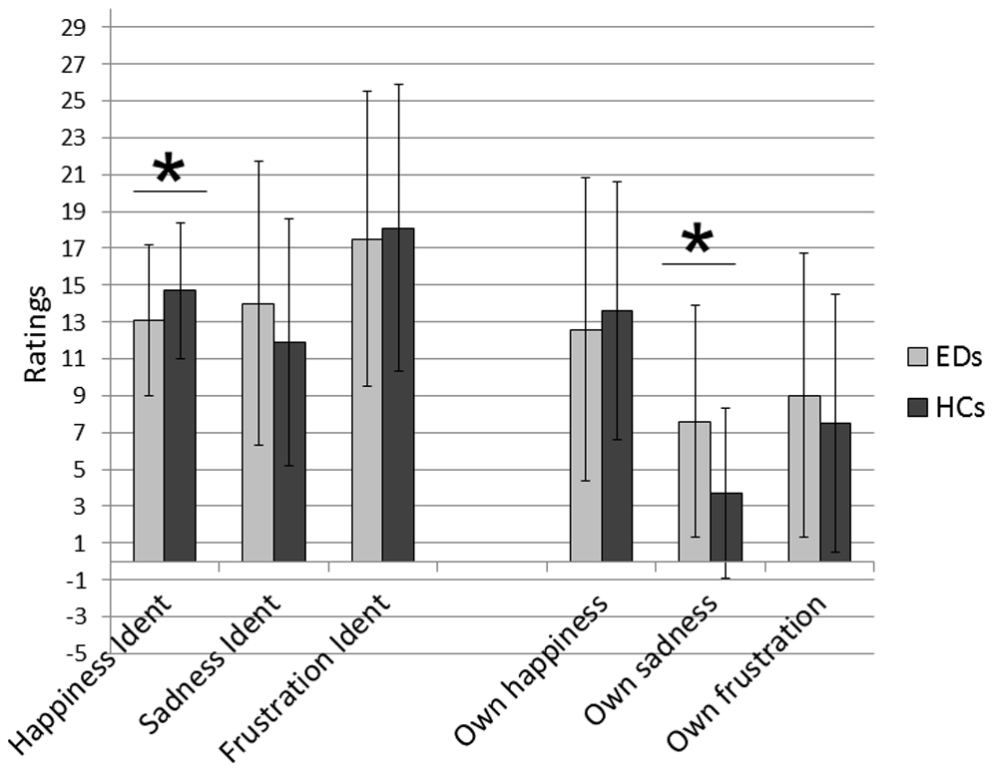
Emotion identification and subjective experience of infant displays. Ratings of positive and negative emotions identified and experienced during the video clips depicting happy, sad, and frustrated infants by participants with Eating Disorders (EDs) and Healthy Controls (HCs). Means and standard deviations displayed. Significant results identified with the asterisk.

Overall, subjects with EDs and HCs reported similar congruent ratings (i.e. video clip displaying happiness rated as positive; video clips displaying sadness and frustration rated as negative; Group: *F* (1, 115) = 003, *p* = .9), but showed significantly different reactions to specific emotions [Group x Emotion: *F* (2, 115) = 4.3, *p* = .01]. The EDs group reported significantly lower positive ratings in response to the happy display [*t* (116) = 2.3, *p* = .02; *ES* = 0.4]. They also reported lower negative ratings of the video clip displaying frustration [*t* (116) = 1.0, *p* = .9; *ES* = 0.1] and higher negative ratings for the video clip displaying sadness [*t* (115) = −1.5, *p* = .1; *ES* = 0.3], but these differences did not reach significance. Emotion identification scores are shown in table 2 and figure 1.

### Subjective experience of positive and negative emotions

There were no overall differences between the EDs and HCs groups in the ratings of positive and negative emotions experienced during the video clips [Group *F* (1, 115) = 1.9; *p* = .2]. However, EDs and HCs showed significant differences in the response to specific emotions [Group x Emotion: *F* (2, 115) = 6.4, *p* = .002]. Subjects with EDs reported significantly higher negative ratings in response to the video clip displaying a sad infant [*t* (115) = −3.8, *p*<.0001; *ES* = 0.7]. They also showed higher negative emotional reactivity to frustration [*t* (116) = −1.2, *p* = .2; *ES* = 0.2] and lower positive emotional reactivity to happiness [*t* (116) = .8, *p* = .4; *ES* = 0.1], but these differences did not reach significance. Emotional responses scores are shown in table 2 and figure 1.

### Film task: facial expressions to positive and negative emotions

**Figure 2:**
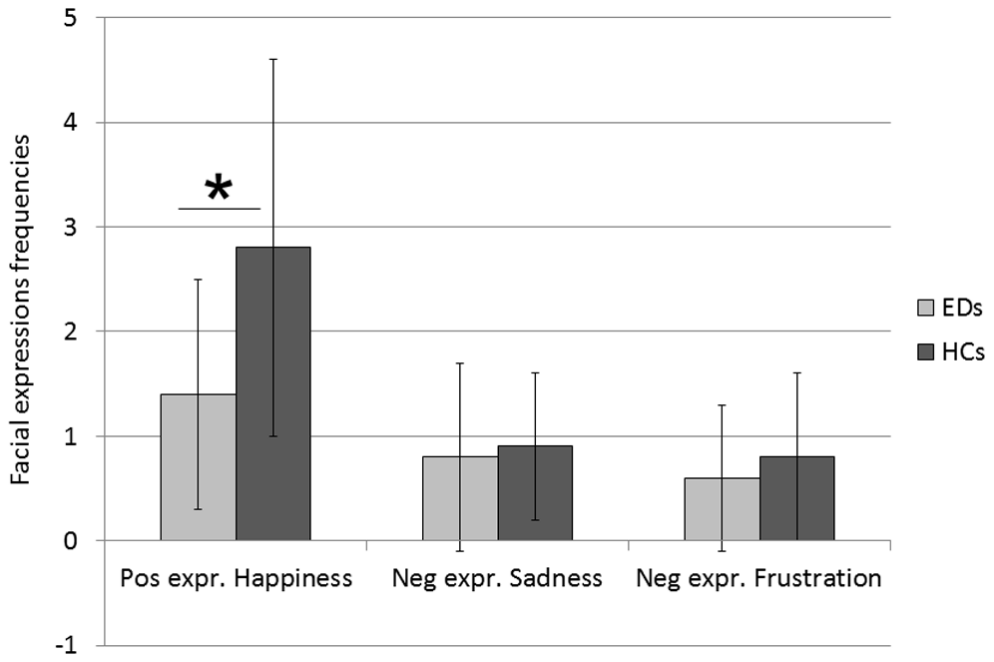
Facial expressions to positive and negative infant displays. Frequencies of facial expressions emitted in response to the video clips depicting happy, sad, and frustrated infants in people with Eating Disorders (EDs) and Healthy Controls (HCs). Means and standard deviations displayed. Significant results identified with the asterisk.

Overall, subjects with EDs displayed fewer emotional expressions in response to the video clips than HCs [Group: *F* (1, 105) = 15.8, *p*<.0001]. The largest difference was observed for the frequency of positive facial expressions to the video clip displaying a happy infant [Group x Emotion: *F* (2, 105) = 13.1, *p*<.0001; *t* (109) = 4.9, *p*<.0001; *ES* = 0.9]. Participants with an ED also had fewer negative expressions in response to the video clips displaying frustration [*t* (107) = 1.2, *p* = .2; *ES* = 0.1] and sadness [*t* (107) = 1.7, *p* = .09; *ES* = 0.2], but these differences did not reach significance. Frequencies of facial expressions are shown in table 2 and figure 2.

### Film task: frequency of looking away

No significant differences between groups were found in the frequencies of looking away from the emotional displays [Group: *F* (1, 105) = 2.3, *p* = .1; Group x Emotion: *F* (2, 105) = .7, *p* = .5]. However, subjects with EDs looked away more often overall than HCs (Happiness: *ES* = 0.2; Frustration: *ES* = 0.3; Sadness: EDs *ES* = 0.2). Frequencies of looking away are presented in table 2.

### Correlations between behavioural measures and eating disorders symptoms and depression scores

The frequency of positive facial expressions displayed in response to the happy infant were negatively correlated with the attachment insecurity ratings in the ED group [*r* (38) = −.6, *p* = .009], but did not correlate with severity of ED symptoms [EDE-Q total subscale: *r* (38) = −.05, *p* = .8) or with Depression subscale scores on the DASS [*r* (38) = −.4, *p* = .1].

The intensity of negative emotions experienced in response to the sad infant video clip also correlated with attachment insecurity scores [*r* (38) = .5, *p* = .03], but not with ED symptoms severity [*r* (38) = .03, *p* = .8] or depression scores [*r* (38) = .2, *p* = .1].

## Discussion

The aim of this study was to investigate emotional processing of infant cues in people with EDs compared to HCs. Findings showed no significant differences between groups in the attentional responses to happy and sad infant photographs. However, there was a tendency to disengage from happy infants in the ED group. People with EDs rated infants' happiness less intensively than HCs and reported greater negative emotional reaction to the sad infant display. Finally, patients had a reduced positive facial mimicry in response to the happy infant video clip. Insecure attachment was negatively correlated with positive facial expressions displayed in response to the happy infant and positively correlated with the intensity of negative emotions experienced in response to the sad infant video clip.

### Attentional bias to happy and sad infants

The finding that smiling infant faces did not produce a stronger attentional bias in healthy young women was somewhat surprising, as it is generally thought that positive infant faces are highly salient for humans [11]. However, the literature examining responses to infants' stimuli suggests that this process is complex and might be particularly affected by motherhood, with mothers being more sensitised to detect and respond to infants' signals than non-mothers [17]. An interesting series of studies examined attentional processes in women in early and late pregnancy and in the post partum period, highlighting a difficulty to disengage attention from distressed infants [33], [34]. Pregnant women with high levels of depression did not show this bias [12], but following a course of Cognitive-Behavioural Therapy and reduction in depressive symptoms vigilance towards infant distress occurred [13]. None of the participants included in our study were pregnant and less than 10% of the sample reported to have children. This might have contributed to the blunted attentional response to infants' faces found in our study and the lack of differences found between our clinical and non clinical samples.

### Emotional identification of and subjective experience of infant displays

Lower positive ratings of the happy infant display in patients with EDs are consistent with findings of abnormal social hedonic processing in this clinical population [18]. Sensitivity to reward in EDs seems to be domain-specific. Recent experimental data, for example, indicate that pictures showing extreme emaciation and physical exercise are perceived positively by people with AN and are associated with reduced startle response (i.e. appetitive response) and increased attentional engagement compared to HCs (emaciation [35]; physical activity [36]).

The higher negative ratings of the sad infant suggest that there might be high levels of empathy for sadness in EDs, which is consistent with work showing vicarious distress to others suffering [37] and normal levels of affective empathy in AN [38].

### Facial expressions to infant displays

Impaired facial expressivity in EDs was found particularly in response to happy infants in this study. This is in line with previous findings, indicating decreased expressivity in response to film clips of adults [9] and scenes depicting comedy and tragedy [10] in EDs. Impaired facial affect in EDs is also evident in non-social contexts. For example, people with AN show significantly less facial expressions of anger/frustration and joy than controls while playing a video game [39], and also less facial expression to food pictures [40]. The finding that people with EDs showed reduced facial expressivity in response to happy infants contributes to the understanding of the quality and extent of impaired reward sensitivity in this population (i.e. infants' faces are considered to be more salient than adults faces).

It is possible that this style of reacting may stem from maladaptive core beliefs about the value of experiencing emotions [41], [42]. An alternative hypothesis is that biological changes associated with starvation, such as low dopamine function, may contribute to this effect. In this study, impaired facial affect in response to happy displays was associated with attachment insecurity and may represent a learned, maladaptive emotional regulation strategy characterized by avoidance and suppression of positive emotions.

### Limitations

The major strength of the study is the use of experimental measures to explore social emotional processing of infant cues in EDs. Nevertheless there are several limitations. The sample size of participants with BN is small. For some aspects of the study (i.e. questionnaires and facial expressions recording) there were missing data. In the visual probe detection task only two set of emotional stimuli, rather than three, were presented (happy and sad infants). This was because we used a standardised set of stimuli which do not include infant anger/frustration. The measurement of facial affect involved expert coding. Although we had high measures of inter-rater reliability, it is possible that a form of automatic machine learning or a more sophisticated rating measure may have reduced the variance. We used novel film clips to depict emotional expressions.

### Implications

Our findings showed that people with EDs have impaired facial reactivity to, and lower positive ratings of, happy infant displays. Abnormal reward sensitivity for social stimuli and maladaptive emotional regulation strategies, such as avoidance and suppression, might underlie this feature. The understanding of basic cognitive and perceptual processing of social stimuli might strengthen the theoretical foundation and effectiveness of treatments targeting interpersonal difficulties in EDs, such as interpersonal psychotherapy.

## Conclusions

People with EDs do not have marked abnormalities in the perceptual processing of infant faces. However, they do have attenuated emotional responsivity and facial mimicry in response to infant happiness. The understanding of these processes might improve the theoretical foundation and effectiveness of treatments targeting interpersonal functioning in EDs.

## Supporting Information

Study database S1 (SAV)
